# Medical Opinion and Movement

**Published:** 1908-07-25

**Authors:** 


### Medical Opinion and Movement.

Dr. M. B. Ebbell lays stress on the fact that rickets is almost unknown in regions near the Equator, and increases in frequency from the Equator towards the North. These geographical variations, he contends, do not depend upon racial differences. In the north of the United States, for instance, Italians and negroes pay a much heavier tribute to the disease than they do in their own southern climes. In Russia, according to Dr. Kissel, who has studied the disease in different latitudes, it is found to diminish steadily as one passes from Moscow to the Crimea. From these facts he draws the conclusion that deficient sunlight plays an important *rôle* in the causation of rickets. In further support of this view he points out that the most severe forms of rickets occur in the spring time, in consequence, as he would adduce, of the lack of the sun's rays during the previous winter. Moreover, the disease is more common among the poor in the towns than in the country, or among the poor altogether than the more well-to-do classes, who are able better to provide fresh air and sunlight for their children. On the other hand, the fact that gastro-intestinal troubles of infants belong rather to the summer months is contrary to the usually accepted connection between rickets and disorders of digestion.

A more plausible theory has been put forward recently by Dr. Leonard Findlay. He suggests —and supports his suggestion by some interesting experiments on animals—that an important ætiological factor in the causation of rickets is confinement, or the lack of exercise and free movements allowed to infants. By feeding puppies on a diet deficient in proteid and fat, such as would be considered likely to produce rickets, he is unable to produce the disease. The disease is, however, easily developed by confinement, although a proper diet be prescribed. Control animals fed on the same food, but allowed free exercise, do not develop the disease. Among the many theories and views that have been put forward, lack of fresh air and sunlight have generally been held as contributing factors, but the actual condition of confinement, involving deficient exercise and movement of the infant's limbs, has not received much consideration. Popular ideas and maternal instincts have often to be corrected by more accurate scientific knowledge; sometimes, however, they are corroborated, and it is worthy of note, and should be placed to their credit, that many mothers of the past generation have expressed their disapprobation of the modern use of the cot and perambulator, in which the child lies day and night, with restrained limbs. Without depreciating the importance of the dietetic factor, the view put forward by Dr. Findlay is very suggestive and there is much to recommend and support it. The geographical distribution of the disease falls naturally into line with it, and many other facts, such as the incidence of the disease in the early part of the year, among the poor, and among the townspeople. It suggests, too, that valuable as fresh air undoubtedly is, it would be better for the infant to lie kicking on the floor of a well-ventilated room than to be taken out confined in a narrow perambulator.

In a recent issue we gave the experiences of an eminent French surgeon in the employment of Bier's hyperæmic method of treatment, and we commented on the fact that the method has as yet found little favour in this country. Since then an address has appeared on the subject by Mr. H. F. Waterhouse, which shows that he is quite as enthusiastic on the method as observers on the Continent, and confirms in every respect the claims that have already been made for this form of treatment. He cites several cases of remarkably rapid cure in every variety of acute and chronic local inflammatory condition, and has found the treatment especially efficacious in cases of tuberculous joints, combined with injections of 10 per cent. iodoform glycerine emulsion. Wherever possible Mr. Waterhouse uses elastic constriction by a short Martin's bandage, 2 to 2½ inches in breadth. The degree of constriction is regulated and controlled by pulsation in the artery beyond constriction, and by the feelings of the patient. The bandage should cause no pain to the patient. On the contrary, in acute inflammations the proper application of the bandage is almost invariably accompanied by a considerable diminution in pain. In affections of the head and neck, Mr. Waterhouse constricts the vessels of the neck by means of an ordinary garter elastic band. For parts where the elastic band is inapplicable the cupping glass is used, and this is applied every alternate five minutes for an hour twice a day.

In the early stages of an organic nervous disease the Babinski sign has been found of the greatest possible service in assisting to exclude functional diseases. Mendel's reflex, which was made known to the profession some three or four years ago, is not so well known. It has been specially studied by Dr. O. B. Meyer, and he finds it of even greater value than the Babinski sign, inasmuch as he has been able to evoke it when the Babinski sign is negative. Mendel's reflex is elicited by placing the foot with its inner surface on a firm basis and percussing the dorsal tendons. In normal individuals percussion causes a dorsal flexion of the second to the fifth toes, but with certain organic changes of the nervous system percussion results in a plantar flexion. Dr. Meyer finds that dorsal flexion obtains in all cases of functional affections and in tabes dorsalis, but in other organic affections the plantar reflex results. He has met with ten cases recently in which the Babinski sign was negative but Mendel's reflex was plantar. In one case of complete paraplegia of all four limbs, all the signs and symptoms pointed to a hysterical condition; Mendel's sign alone pointed to an organic lesion. The case was subsequently shown to be one of encephalitis pontis.

"PHOSSY JAW" is not a common complaint in England, and by far the larger number of students qualify and practise without ever having had an opportunity of seeing a case of this condition. Yet old practitioners can easily call to mind the time when the disease was by no means so rare as it is to-day. What legislation has done for the "match maker" in England it has similarly effected in his interests in Germany, Holland and France, though in a lesser degree, because the trade in phosphorus in these countries is relatively larger than with us, while the class of workman is—with the exception of Germany—relatively poorer and more unhygienic. For these reasons phosphorus poisoning is still to be met with, as a rarity, in Continental hospitals, while students there have splendid opportunities of studying the still very obscure pathology of the disease. Nowhere, however, can it be said to be so frequent and so destructive as in Austria. There the factories still employ the "white phosphorus"; their workmen are still submitted to dangers similar to those with which the English factory labourer had to cope with thirty years ago. In one province alone—that of Styria—not less than fifty to sixty cases of severe phosphorus necrosis are recorded yearly, while a much larger number of slightly affected patients are annually treated for the same complaint.

THIS frequency of phosphorus poisoning in Austria is due to practically the same causes which led to the mortality in England before the legislature stepped in to protect the phosphorus worker. Insanitary and badly-ventilated working rooms, the use of the toxic phosphorus of the white and yellow varieties, unusually long hours, and the large amount of material used are responsible for much of the severity of these cases. But something must also be debited to the prevalence of tuberculosis. There is probably no European country that can rival Austria in its tuberculosis statistics, and as in Germany it is stated that in the large cities ninety per cent. of children are rachitic, so it is no exaggeration to state that in the larger Austrian centres the same percentage of inhabitants suffer from tuberculosis in some form or other. Leaving undiscussed for the moment the question whether or not phossy jaw is a tuberculous necrosis, changed and modified by phosphorus poisoning—a theory that has as much against as for it—the fact that tuberculous persons are much more sensitive to the effects of phosphorus fumes is now generally admitted, even by those who deny a connection between the two conditions. The lowered vitality of a large percentage of factory workers is primarily responsible for these terrible statistics furnished by the Austrian phosphorus factories, and the whole question of prevention and prophylaxis is at present being earnestly considered by the Austrian authorities. The opposition to any legislative measures of reform there is as great as it was with us when legislation was first introduced. All the old arguments that such legislation will kill the trade, that toxic phosphorus must be used, and that the prevalence of the disease is due to the ineradicable stupidity and obstinacy of the workers themselves, are brought forward, and a strong opposition to the new acts, which have for their object the improvement of the abominable conditions under which the worker in the Austrian phosphorus factories at present lives, has been organised. Nevertheless, the Government seems at last to have grasped the importance of dealing with the matter, and there can be little doubt that Austria will shortly possess similarly excellent laws to those which in other countries prohibit the exposure of a class of workman to what are really preventable and removable dangers.

THERE are two observations of considerable interest communicated to the current number of the very ably conducted *Journal of the Royal Army Medical Corps*. Capt. Harding finds that in a small proportion of soldiers an aortic systolic murmur can be produced by the act of throwing back the shoulders with the arms raised. This peculiarity was observed in perfectly healthy men, in whom it could be evoked in no other way. It is suggested that this curious artefact may explain how it is that of men invalided home from abroad for V.D.H. (valvular disease of the heart) a certain number are placed on the duty list almost as soon as they arrive in England. It is supposed that the act of holding up the folded shirt so as to expose the præcordial region for examination is thus responsible for errors in diagnosis.—Major Smith advances an ingenious speculation as to the causation of twins based on the discovery that all the spermatozoa in the semen of a man whose wife regularly bore twins were double headed. As the writer truly says, the subject is a difficult one, and has intimate bearings on the question of heredity; he is even a little inclined to the inference that the usual view of a spermatozöon as a mere fertiliser of an ovum may be wrong, and that the former may be the more important agent of the two in the constitution of the embryo. Major Smith is at least entitled to credit for a most remarkable observation, and for publishing it.

DR. R. HOWARD, of British Central Africa, agrees with Manson and Daniels that there is a definite and well-defined tertiary stage in yaws, a point which has been a good deal in dispute among those who have had experience in this disease. The text-books are all very vague on the subject still, so every item of fresh evidence is valuable. Dr. Howard lives in a comparatively low-lying region about the south end of Lake Nyasa, where yaws is plentiful, and syphilis and tubercle are rare. In the neighbouring highlands, where Arab and European contact with the natives has chiefly occurred, the reverse is the case; syphilis is not at all uncommon, whereas yaws is. It is quite certain that within recent years intercommunication has vastly increased among the natives, owing to their developing habits of travel in search of work, and this mal-propagation of syphilis is held to favour the idea that frambœsia, which is, of course, often contracted in childhood, protects to some extent against that disease. In the lowlands lesions curable by iodides are much more frequent than the incidence of primary and secondary syphilis will account for, and it is especially noted that most of the patients admit a previous attack of yaws, but deny syphilis; some of them are children of tender years, and the probability seems to be that most of them are cases of tertiary yaws. The usual types are: (1) skin lesions characterised by chronic infiltration and ulceration; these, from the description, seem to resemble syphilitic ulcers fairly closely; (2) lesions of bones and joints, especially chronic synovitis of the larger joints, nodes of localised periostitis, and general diffuse osteitis accompanied by intense nocturnal pain and (3) destructive ulceration of the nose, palate, and pharynx. The author admits the difficulty in diagnosing these lesions from those of syphilis, which is all the greater in that potassium iodide seems to be the best treatment for them. He feels convinced that there really is this tertiary stage in yaws, and records also his impression that its lesions are more prone to spontaneous resolution than is the case with syphilis. His paper is published in the *Journal of Tropical Medicine and Hygiene.*

---

CERTAIN areas of abdominal cutaneous hyperalgesia have been described as associated with appendicitis, notably by Mr. Sherren in 1903. These are considered in the *Quarterly Journal of Medicine* by Dr. Robinson, who arrives at conclusions which to some extent support the previous researches. Thus he inclines to the view that a band or triangle of cutaneous tenderness in the territory of the eleventh right dorsal segmental nerve indicates appendicitis almost, though not quite, with certainty. Such tenderness he finds in 21 per cent. of all cases of appendicitis, whereas Sherren found it in 32 per cent. The clinical significance of this symptom has been variously regarded by different observers: Sherren regards it as a sure sign that the appendix is not yet perforated or gangrenous, while other authorities have held it to be a sign of grave complications such as peritonitis, subphrenic abscess, pneumonia, and so on. Dr. Robinson, after carefully weighing up the pros and cons, concludes that cutaneous tenderness has very little bearing on the prognosis or treatment, though he finds it rather less frequent in abscess cases than in others. General peritonitis may develop and abscesses may form, notwithstanding the presence of abdominal cutaneous hyperæsthesia: indeed the author believes that in some cases of intense and widespread superficial tenderness it is the inflammation of large areas of the peritoneum rather than that of the appendix which excites the reflex. This is a point on which the authorities are at variance, though it might be thought that in connection with so common a disease it would have been cleared up long ago. Perforated duodenal ulcer, renal colic, perimetritis, and intestinal colic are some of the conditions in which tenderness of the skin of similar distribution to that caused by appendicitis has been found.

THE actual inspection of the various mucous membranes is an aid to diagnosis which has been very largely extended by the joint labours of surgeons and instrument makers in recent years. It is now claimed by Messrs. H. S. Souttar and Theodore Thomson that direct inspection of the gastric mucous membrane is feasible by means of a new instrument which they have devised. In overcoming the great difficulties which the problem of direct inspection of the stomach presents, they have combined in one instrument the advantages of the inventions of Mikulicz and of Kelling, and yet have avoided the objections which attended the use of both those instruments. With this gastroscope they have examined twelve patients. In eight the appearances were those of normal mucous membranes; and in the other four various abnormalities were recognised, including carcinoma of the pylorus in one case and gastritis in another. It is possible by means of a rotating nose-piece to inspect in turn the whole interior of the stomach, with the exception of a small patch about the cardiac orifice, and it is claimed that this can be done entirely without risk. Anæsthesia is not absolutely necessary, as is proved by the three photographs of a patient taken during and after introduction, each plate having been exposed about eight seconds: this man, however, had been in the habit of washing out his own stomach, and was no doubt exceptionally tolerant.

---

RESEARCHES into the excretion of creatinin in parturient women have suggested to Dr. C. N. Longridge a new conception of the process of uterine involution, which he tentatively puts forward in the *Journal of Obstetrics and Gynæcology.* The sarcolactic acid produced by the powerful action of the uterine muscle during delivery he regards as neutralised by the circulating blood. When, however, the uterus is emptied and contracts firmly down the two conditions of relative anæmia and consequent diminished alkalinity are produced, which favour rapidity of autolysis. After four or five days the uterus becomes considerably softer, and blood percolates once more freely through it; thus the sarcolactic acid is more freely neutralised, and therefore involution becomes slower, as it is recognised clinically to do. In considering possible objections to this hypothesis, the author points out that during the early puerperium the lochia are red, and therefore the uterus cannot be supposed to be completely anæmic: he gets over this difficulty by suggesting the possibility that red lochia may be really abnormal, not normal at all. It is remarked upon that in no other mammal are the lochia blood-stained, and also that it is not very uncommon to see entirely bloodless lochia in the human race; this is certainly, as Dr. Longridge says, a recognised clinical fact. On this ingenious supposition he explains a remarkable case of superinvolution recorded thirty years ago, in which the uterus completely disappeared, by suggesting that the circulation never became re-established and that the uterus consequently dissolved away and was discharged in the brown watery fluid which took the place of the normal lochia.

